# Supplementary material for: Eigenmode orthogonality breaking and anomalous dynamics in multimode nano-optomechanical systems under non-reciprocal coupling
Source: Nat Commun. 2018 Apr 11;9:1401. doi: 10.1038/s41467-018-03741-8 (PMC5895839; doi:10.1038/s41467-018-03741-8)
Supplement: Supplementary file 1 — Supplementary Information [file 41467_2018_3741_MOESM1_ESM.pdf]

Supplementary material for
"Eigenmode orthogonality breaking and anomalous dynamics in multimode nano-optomechanical systems under non-reciprocal coupling"

Mercier de Lépinay et al.

SUPPLEMENTARY NOTE 1: Experimental setup and AOM calibration

Experimental setup

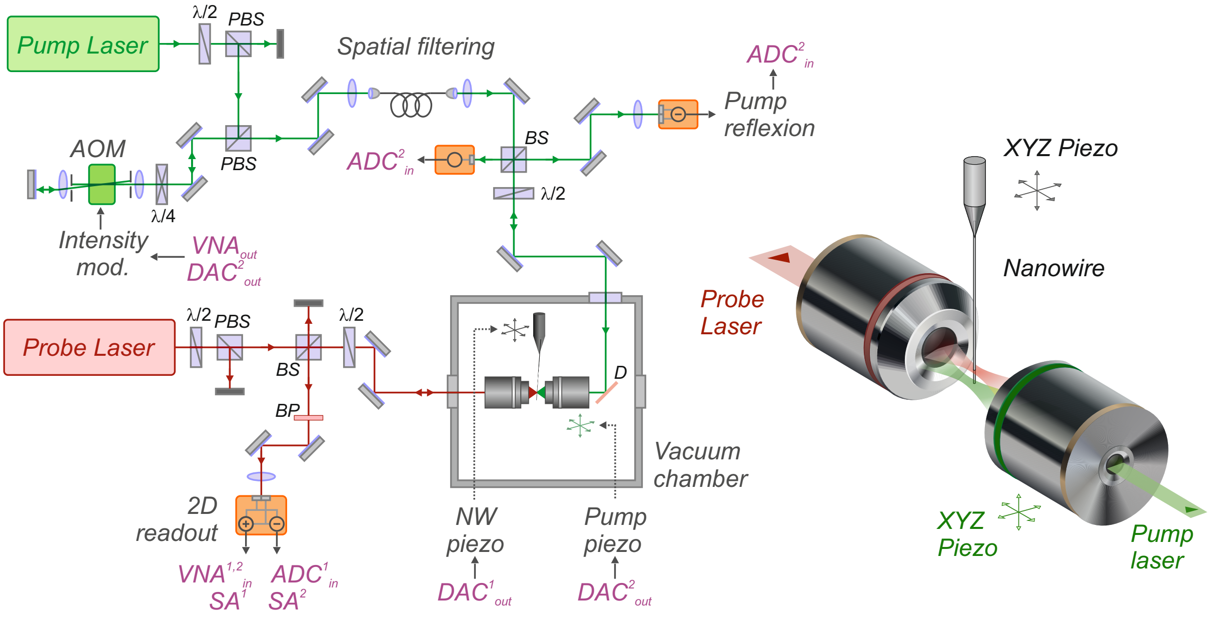

Supplementary Figure 1: **Sketch of the experimental setup and signal routing** . PBS/BS: polarizing/non-polarizing beam splitters, D: dichroic, BP: red band-pass filter. VNA: vector network analyzer, SA^{i}: spectrum analyzers, ADC^{i}_{in}: input channels of the signal processing cards, DAC^{i}_{out}: output channels of the signal processing cards, AOM: acousto-optic modulator. Inside the vacuum chamber, the objective that serves to focus the probe beam if fixed in space, the nanowire is mounted on a XYZ piezo scanner. The microscope objective that serves to focus the green pump laser is also mounted on a XYZ piezo scanner to spatially adjust the optical force field gradient. The NW position is locked in X(Z) directions using the reflected probe difference(sum) signal. The pump laser position with respect to the NW is monitored and controlled on the pump laser reflection. The outputs channels of two digital /analog cards ($DAC^{1,2}_{out}$) are used to control the NW position (3 channels, X Y Z) and pump beam position and static intensity (4 output channels).

Calibration of the green optical power

The green light beam is used to produce a rotational, static force on one hand and a small modulated test force for response measurements on the other hand. Namely, the intensity of the rotational force and the amplitude of the driving force are both controlled by the same Acousto-Optic Modulator (AA OptoElectronics, MT200-A0.5-VIS) used in a double-pass configuration, through a RF driver with a $0-1\,\mathrm{V}$ input command modulation range enabling total extinction of the beam. The AOM response to this modulation command is highly non-linear (see **Supplementary Figure 2**). Therefore, if no particular precaution is taken and a fixed modulated command voltage amplitude is used for various command offsets then the driving force applied on the nanowire will be modified for each command offset, that is, for each intensity of the rotational force.

In order to decouple the intensity of the static rotational force applied on the nanowire and the amplitude of the coherent test force used to explore its mechanical properties, we carefully calibrated the AOM optical response while sweeping the two control parameters (voltage offset and modulation amplitude). This calibration was then used to compute the ensemble of AOM drive amplitudes that would yield a constant force modulation depth for a series of rotational force intensity. For this calibration step as in the experiment the DC offset was set by an analogic output of a DAQ card also used for standard operation of the experiment (positioning, DC readout), while the modulated signal was provided by the output channel of a network analyzer (see **Supplementary Figure 2**). Both outputs were analogically summed and fed to the AOM driver. Contrary to the experiment, the output signal of the AOM was then directly sent on a previously calibrated rapid photodiode of very flat frequency response. The

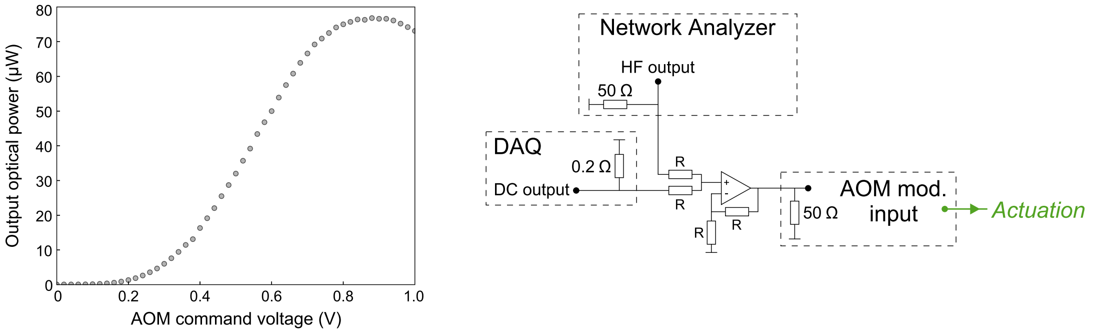

Supplementary Figure 2: **Control of the green optical power** Left: Static transmission curve of the acousto-optic modulator. AOM output light power in response to $0 - 1\,\mathrm{V}$ command voltage. Due to the observed non-linearity, a careful calibration is required in order to simultaneously and independently use the AOM for controlling the mean intensity seen by the nanowire and applying a dynamical optical force excitation. Right: Sketch of the electronic summing module of DC and AC command voltages sent to the AOM. The DAQ was in fact used between 0 and $2\,\mathrm{V}$ to produce a $0 - 1\,\mathrm{V}$ offset on the AOM, due to the impedance mismatch between the output of the amplifier and the input of the AOM driver. Meanwhile the driving amplitudes taken into account in the present calibration are directly those obtained at the output of the network analyzer.

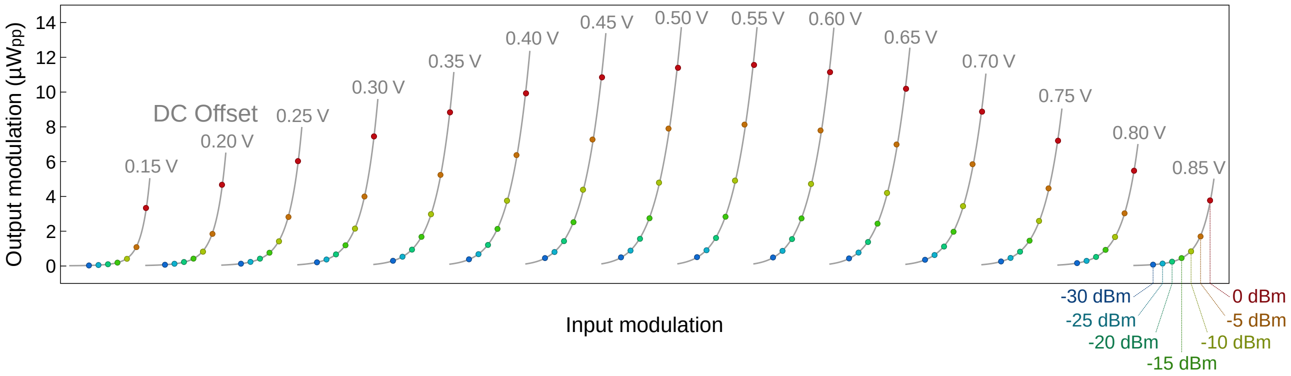

Supplementary Figure 3: Extract of the scan of the 2 parameters of the AOM command. For each applied voltage, the output light modulation was measured for a series of input voltage modulations, which are labeled on the right data set.

modulation frequency used for the calibration was swept in the region of the mechanical frequencies to reproduce the experimental situation. The resulting output modulation amplitude was recorded by the network analyzer, averaged over frequencies (after having checked that the frequency dependency was negligible) and calibrated in terms of light power (see **Supplementary Figure 3**).

For each scanned voltage offset, a polynomial fit was applied on the measured series of modulation amplitudes. A 2D interpolation of these fits was then used to generate a grid of calibration points, that can be represented as an abacus (see Supplementary Figure **Supplementary Figure 4**). Finally, the measurement interface program directly used this grid of points to calibrate the modulation depth generated by the network analyzer with respect to the offset voltage such that the test force amplitude would stay at the constant over the variation of the rotational force intensity. This automatic numerical operation corresponds to calculating the modulation depths on a horizontal line in the abacus reproduced on **Supplementary Figure 4**). During the experiment the driving force amplitude was kept at a relatively low amplitude so that it is reasonable to assume that the nanowire is mainly sensitive to the local value of the resonant driving force and not to its spatial derivatives (and in particular not to its possible rotational component). This also ensures that the DC light power is not significantly modified by the modulation even in non-linear regions of the AOM. The rotational force series presented in Figure 2 of the manuscript and this presented in **Supplementary Figure 5** and **Supplementary Figure 6** were for example obtained with a light power modulation amplitude of $1\,\mu\mathrm{W}$ peak-to-peak (see red dashed line on **Supplementary Figure 4**), which was measured to correspond to $270\,\mathrm{aN}$, and this for all light power offset levels from $2\,\mu\mathrm{W}$ to $77\,\mu\mathrm{W}$. The whole calibration

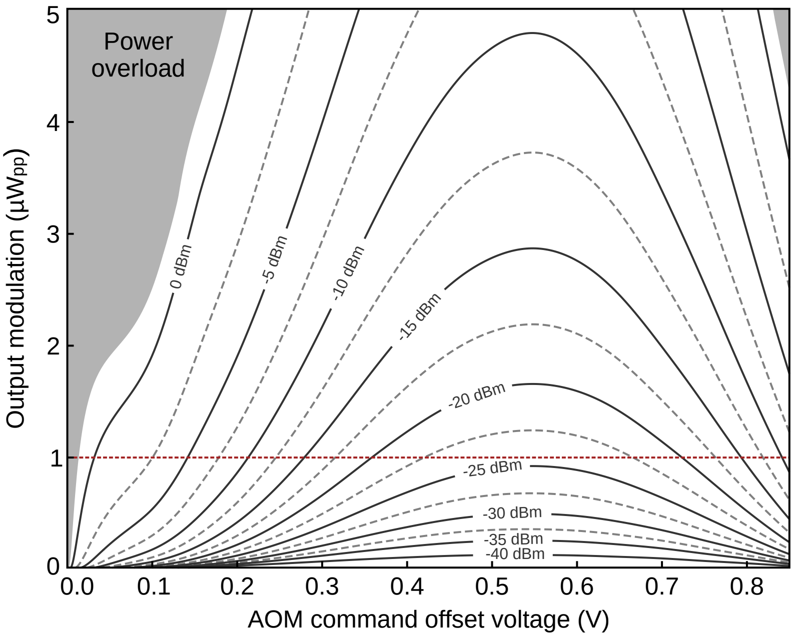

Supplementary Figure 4: **Abacus of the output light power modulation obtained for each command DC voltage.** The driving force amplitude can be maintained constant while varying the static rotational intensity (through the DC command voltage) by applying the command modulations crossed by a horizontal line – like the red dashed line corresponding to $1\,\mu W_{pp}$ whatever the offset. This abacus was only generated for the operator's convenience as these modulation depths were in fact automatically computed from the calibration grid.

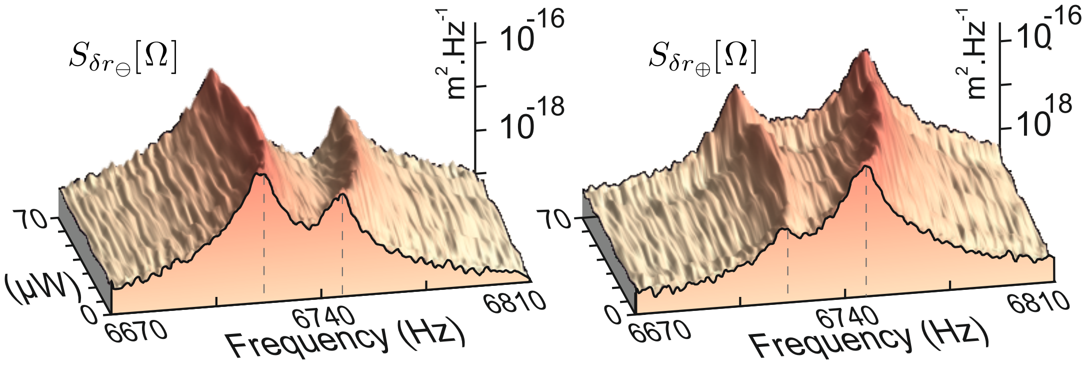

Supplementary Figure 5: **Analog of Fig.1e, on the right side of the optical axis (↻).** In this region of opposite rotational, the frequency splitting increases with the optical pump power.

can obviously be rescaled by a global factor if the AOM input light power is modified for the needs of the experiment.

SUPPLEMENTARY NOTE 2: Measurements on the opposite side of the waist ↻

Supplementary Figure 5 and **Supplementary Figure 6** show the measurements realized on the other side of the optical axis, following the same progression and conventions as in the manuscript.

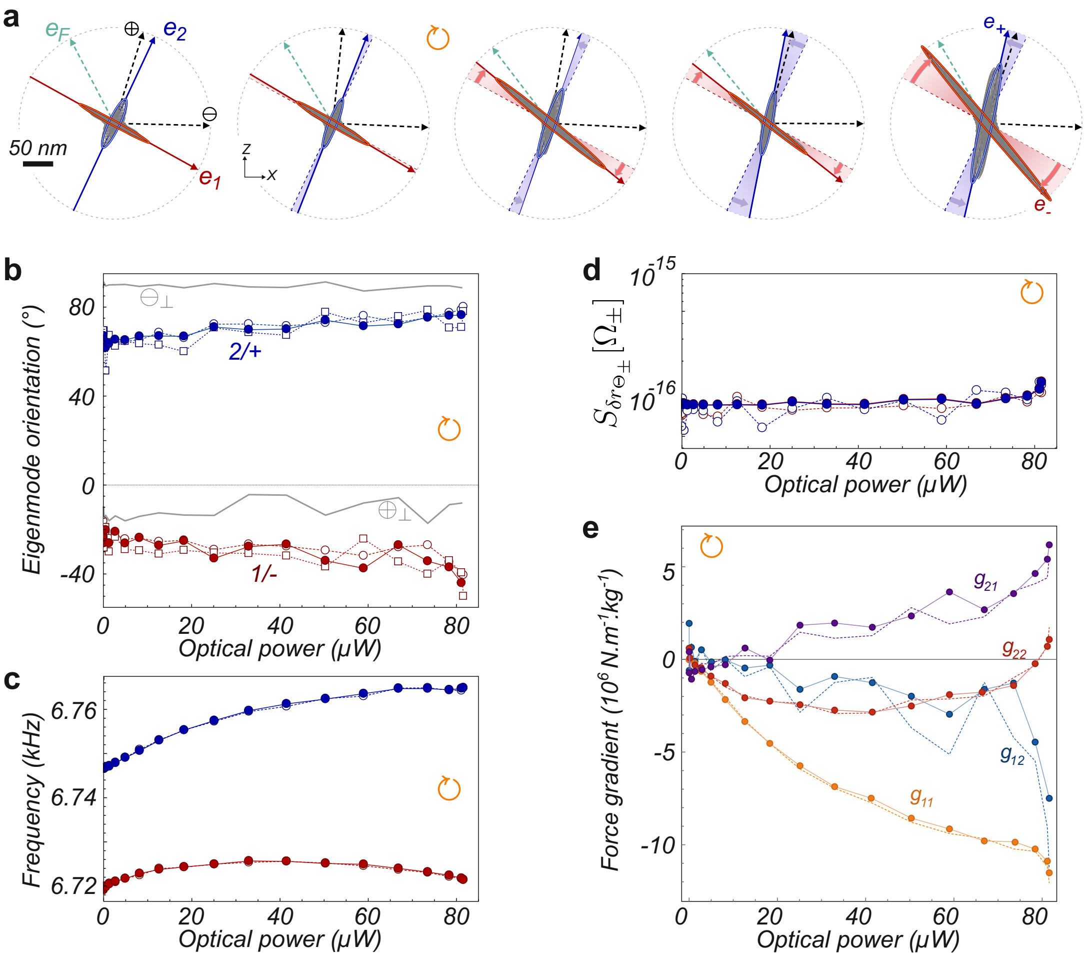

Supplementary Figure 6: **Analog of Fig.2 with the same conventions, on the right side of the optical axis (↻)**

SUPPLEMENTARY NOTE 3: Model

Nanowire dynamics in presence of an external force field

Following the dynamical equations exposed in the manuscript, the dynamics of the nanowire can be written in Fourier space:

$$\boldsymbol{\delta r}[\Omega] = \bar{\chi}[\Omega] \cdot \delta \mathbf{F}_{\mathrm{th}} \tag{1}$$

where the susceptibility matrix $\bar{\chi}[\Omega]$ is, in the $e_{1,2}$ basis:

$$\bar{\chi}[\Omega] = \frac{1}{\Xi[\Omega]} \begin{pmatrix} \Omega_2^2 - \Omega^2 - i\Gamma\Omega - g_{22} & g_{21} \\ g_{12} & \Omega_1^2 - \Omega^2 - i\Gamma\Omega - g_{11} \end{pmatrix} \tag{2}$$

where

$$\begin{aligned} \Xi[\Omega]/M_{\mathrm{eff}} &\equiv (\Omega_1^2 - \Omega^2 - i\Gamma\Omega - g_{11})(\Omega_2^2 - \Omega^2 - i\Gamma\Omega - g_{22}) - g_{12}g_{21} \\ &= (\Omega_-^2 - \Omega^2 - i\Gamma\Omega)(\Omega_+^2 - \Omega^2 - i\Gamma\Omega) \end{aligned} \tag{3}$$

where we used the eigenfrequencies:

$$\Omega_\pm^2 \equiv \frac{\Omega_{1\|}^2+\Omega_{2\|}^2}{2} \pm \frac{1}{2}\sqrt{\left(\Omega_{2\|}^2-\Omega_{1\|}^2\right)^2+4g_{12}g_{21}} \tag{4}$$

with $\Omega_{i\|}^2 \equiv \Omega_i^2 - g_{ii}$. The corresponding eigenmodes are

$$\mathbf{e}_- = \frac{1}{\sqrt{g_{12}^2+(\Delta\Omega_\perp^2)^2}}\begin{pmatrix}\Delta\Omega_\perp^2\\ g_{12}\end{pmatrix},\ \ \mathbf{e}_+ = \frac{1}{\sqrt{g_{21}^2+(\Delta\Omega_\perp^2)^2}}\begin{pmatrix}-g_{21}\\ \Delta\Omega_\perp^2\end{pmatrix} \tag{5}$$

where we have introduced :

$$\begin{aligned}\Delta\Omega_\perp^2 &\equiv \Omega_{2\|}^2-\Omega_-^2 = \Omega_+^2-\Omega_{1\|}^2\\ &= \tfrac{\Omega_{2\|}^2-\Omega_{1\|}^2}{2}+\tfrac{1}{2}\sqrt{\left(\Omega_{2\|}^2-\Omega_{1\|}^2\right)^2+4g_{12}g_{21}}\end{aligned} \tag{6}$$

which takes positive values in conservative force fields ($g_{12}=g_{21}$), but can become negative in non conservative force fields. The eigenmode orientations $\theta_\pm$ with respect to the $\mathbf{e_1}$ axis are:

$$\tan\theta_- = \frac{g_{12}}{\Delta\Omega_\perp^2}, \qquad \tan\theta_+ = -\frac{\Delta\Omega_\perp^2}{g_{21}}, \tag{7}$$

The scalar product of coupled eigenmodes is proportional to the 2D rotational of the force field:

$$\mathbf{e}_-\cdot\mathbf{e}_+ = \frac{\Delta\Omega_\perp^2}{\sqrt{g_{12}^2+(\Delta\Omega_\perp^2)^2}\sqrt{g_{21}^2+(\Delta\Omega_\perp^2)^2}}(g_{12}-g_{21}) \tag{8}$$

Their vectorial product amounts to:

$$\mathbf{e}_-\wedge\mathbf{e}_+\big|_y = \frac{g_{12}g_{21}+(\Delta\Omega_\perp^2)^2}{\sqrt{g_{12}^2+(\Delta\Omega_\perp^2)^2}\sqrt{g_{21}^2+(\Delta\Omega_\perp^2)^2}} \tag{9}$$

which is equal to 1 in a conservative force field.

Weak coupling regime

At first order in the external force field gradients, we have:

$$\tan\theta_- \approx \frac{g_{12}}{\Omega_2^2-\Omega_1^2}, \qquad \tan\theta_+ \approx -\frac{\Omega_2^2-\Omega_1^2}{g_{21}}, \tag{10}$$

and for the eigenfrequencies:

$$\Omega_-^2 \approx \Omega_1^2 - g_{11}, \qquad \Omega_+^2 \approx \Omega_2^2 - g_{22}, \tag{11}$$

Strong coupling regime

When the force field gradients dominates the NW intrinsic properties, then eigenmodes converge towards $\mathbf{e}_\pm^\infty$ whose expression is given by:

$$\mathbf{e}_-^\infty = \frac{1}{\sqrt{g_{12}^2+(\Delta\Omega_\perp^{2\,\infty})^2}}\begin{pmatrix}\Delta\Omega_\perp^{2\,\infty}\\ g_{12}\end{pmatrix},\ \mathbf{e}_+^\infty = \frac{1}{\sqrt{g_{21}^2+(\Delta\Omega_\perp^{2\,\infty})^2}}\begin{pmatrix}-g_{21}\\ \Delta\Omega_\perp^{2\,\infty}\end{pmatrix} \tag{12}$$

where $\Delta\Omega_\perp^{2\,\infty} \equiv \frac{1}{2}\left(g_{11}-g_{22}+\sqrt{(g_{11}-g_{22})^2+4g_{12}g_{21}}\right)$ and the corresponding angles $\theta_\pm^\infty$ with respect to $\mathbf{e_1}$are given by:

$$\tan\theta_-^\infty = \frac{g_{12}}{\Delta\Omega_\perp^{2\,\infty}}, \qquad \tan\theta_+^\infty = -\frac{\Delta\Omega_\perp^{2\,\infty}}{g_{21}}, \tag{13}$$

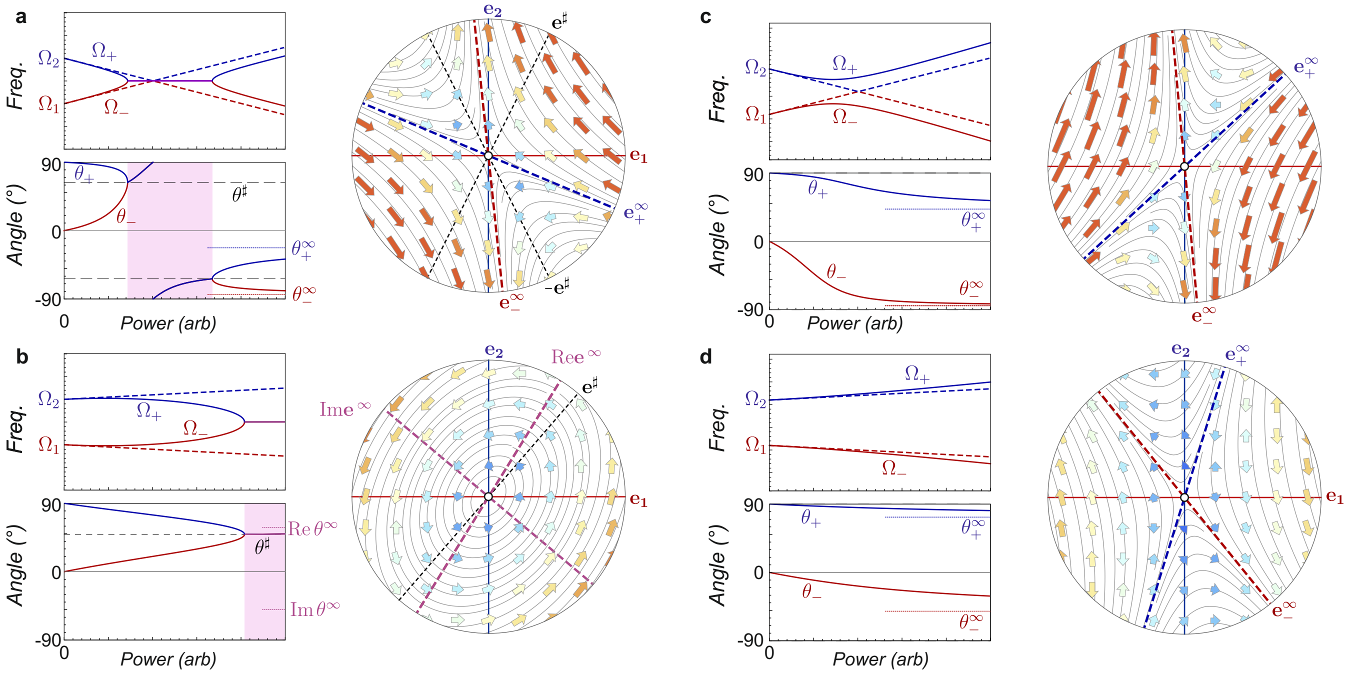

Supplementary Figure 7: **Influence of non-conservative force field gradients** Each panel shows the evolution with the optical power of eigenfrequencies and eigenmode orientations, as well as the local linearized structure of the force field. The flow of the force field is shown as gray lines. Left panels are presenting a bifurcation, while it is absent in the right panels. Upper/lower panels present longitudinal components (g_{ii}) allowing/preventing mode crossing in absence of shear components. In the frequency shift plots, the dashed lines correspond to the evolution in absence of shear components ($\Omega_\pm^2 = \Omega_{1,2}^2 - g_{11,22}$). In the angle plots, the dotted lines at large powers represents $\mathbf{e}_\pm^\infty$ and are also shown in the force field plot as dashed lines. The black dashed lines represent when existing the bifurcation angles $\theta^\sharp$. The force field gradients $(g_{11}, g_{12}, g_{21}, g_{22})$ are proportional to **a** (-0.05,0.04,-0.01,0.05); **b** (0.01,0.04,-0.03,-0.01) **c** (-0.051,-0.1,-0.01,0.05) **d** (0.01,-0.04,-0.01,-0.01). Case a represents a transitory bifurcation, the NW becomes stable again at large powers: the enter into the bifurcation area is observed at the angle $\theta^\sharp$, while the exit from the bifurcation occurs at $-\theta^\sharp$. Case b illustrates the situation of a transition towards a permanent bifurcation. The force field matrix has complex eigenvalues in that case. Panel c and d represents the non bifurcating situations, with opposite contributions from longitudinal force field gradients.

Non conservative force field

At the bifurcation, both eigenfrequencies and eigenmode orientation converge towards the same value:

$$\Omega^{\sharp 2} \equiv \frac{\Omega_{1\|}^2 + \Omega_{2\|}^2}{2} \tag{14}$$

and we have $\Omega_{2\|}^2 - \Omega_{1\|}^2 = \pm\sqrt{-4g_{12}g_{21}}$. As such the bifurcation angle becomes:

$$\tan\theta^\sharp = \pm\sqrt{-g_{12}/g_{21}}, \tag{15}$$

whose sign is the one of $\Omega_{2\|}^2 - \Omega_{1\|}^2$. In some situations, the bifurcation appears at a given power and disappear at larger powers (due to the polynomial dependence of $(\Omega_{2\|}^2 - \Omega_{1\|}^2)^2 + 4g_{12}g_{21}$ with power), in that case the angles of appararition/disappearance have opposite signs.

2D readout

As explained in the article, both outputs of the dual photodiode amplifier produce a projected displacement:

$$\delta r_\beta \equiv \boldsymbol{\delta r} \cdot \mathbf{e}_\beta \tag{16}$$

where $\mathbf{e}_\beta$ is the projective vector $(\mathbf{e}_\ominus, \mathbf{e}_\oplus)$. The thermal noise spectra are defined according to:

$$\langle \delta r_\beta[\Omega] \delta r_\beta[\Omega'] \rangle = 2\pi\delta(\Omega+\Omega') S_{\delta r_\beta}[\Omega]$$

with the spectral convention $\delta r_\beta[\Omega] \equiv \int_{\mathbb{R}} \delta r_\beta(t) e^{-i\Omega t} dt$.

For a measurement vector forming an angle β with the $\mathbf{e_x}$ axis, we have:

$$\delta r_\beta = (\cos\beta\chi_{11} + \sin\beta\chi_{21})\,\delta F_1 + (\cos\beta\chi_{12} + \sin\beta\chi_{22})\,\delta F_2 \tag{17}$$

with a spectral density calculated for uncorrelated Langevin forces of identical noise spectral density $S_F^{\rm th} = 2M_{\rm eff} k_B T$:

$$S^{\rm mod}_{\delta r_\beta}[\Omega] = \frac{S_F^{\rm th}}{|\Xi[\Omega]|^2} \begin{pmatrix} \cos^2\beta\left((\Omega_{2\|}^2 - \Omega^2)^2 + \Omega^2\Gamma^2 + g_{21}^2\right) \\ +\sin^2\beta\left((\Omega_{1\|}^2 - \Omega^2)^2 + \Omega^2\Gamma^2 + g_{12}^2\right) \\ +2\sin\beta\cos\beta\left(g_{12}(\Omega_{2\|}^2 - \Omega^2) + g_{21}(\Omega_{1\|}^2 - \Omega^2)\right) \end{pmatrix} \tag{18}$$

This expression is used throughout the article to fit the experimental data.

Fluctuation dissipation relation

The fluctuation dissipation relation (FDR) connects the expected thermal noise spectrum to the imaginary part of the mechanical susceptibility obtained from a response measurement realized by measuring along the same measurement vector orientation,(a generic $\mathbf{e}_\beta$ here) when applying a test force aligned with the measurement vector ($\mathbf{e_F} = \mathbf{e}_\beta$) with the same spatial profile. In such a measurement, the driven displacement can be written using Supplementary Equation (17) with a coherent drive ($\delta F_1 = \delta F \cos\theta_F$, $\delta F_2 = \delta F \sin\theta_F$). The susceptibility $\chi_{\beta\beta}$ obtained is then:

$$\chi_{\beta\beta} = \cos^2\beta\chi_{11} + \sin^2\beta\chi_{22} + \cos\beta\sin\beta\,(\chi_{12} + \chi_{21}) \tag{19}$$

and the fluctuation dissipation relation becomes:

$$S^{\rm FDT}_{\delta r_\beta}[\Omega] = \frac{2k_b T}{|\Omega|}\,|{\rm Im}\chi_{\beta\beta}[\Omega]| \tag{20}$$

$$S^{\rm FDT}_{\delta r_\beta}[\Omega] = \frac{S_F^{\rm th}}{|\Xi[\Omega]|^2} \begin{pmatrix} \cos^2\beta((\Omega_{2\|}^2 - \Omega^2)^2 + \Omega^2\Gamma^2 + g_{12}g_{21}) \\ +\sin^2\beta((\Omega_{1\|}^2 - \Omega^2)^2 + \Omega^2\Gamma^2 + g_{12}g_{21}) \\ +\sin\beta\cos\beta(g_{12} + g_{21})(\Omega_{2\|}^2 - \Omega^2 + \Omega_{1\|}^2 - \Omega^2) \end{pmatrix} \tag{21}$$

This expression can be compared to Supplementary Equation (18), they are equal in a conservative force field when ($g_{12} = g_{21}$), but differ in a non-conservative force field. As such our modelization, which perfectly reproduces the measured spectra and responses even when non standard lineshapes are observed already contains a violation of the FDR. This is due to the breaking of the eigenmode orthogonality.

Principle of angular and spectral tomography of the 2D Brownian motion

We investigate here the angular dependence of the spectral densities of the projected displacement noise. To do so we derive Supplementary Equation (18) with respect to the measurement angle, β. The noise power measured at a frequency Ω is maximized when β reaches $\beta_{\rm opt}[\Omega]$ solution of :

$$\tan 2\beta_{\rm opt}[\Omega] = 2\frac{g_{12}(\Omega_{2\|}^2 - \Omega^2) + g_{21}(\Omega_{1\|}^2 - \Omega^2)}{(\Omega_{2\|}^2 - \Omega^2)^2 - (\Omega_{1\|}^2 - \Omega^2)^2 + g_{21}^2 - g_{12}^2}. \tag{22}$$

The extrema of the noise spectral density is:

$$S_{\delta r_{\beta_{\rm opt}[\Omega]}}[\Omega] = \frac{S_F^{\rm th}}{2|\Xi[\Omega]|^2} \left\{ \begin{array}{l} (\Omega_{1\|}^2 - \Omega^2)^2 + (\Omega_{2\|}^2 - \Omega^2)^2 + g_{12}^2 + g_{21}^2 + 2\Gamma^2\Omega^2 \\ \pm\sqrt{\left((\Omega_{1\|}^2 + \Omega_{2\|}^2 - 2\Omega^2)^2 + (g_{12} - g_{21})^2\right)\left((\Omega_{1\|}^2 - \Omega_{2\|}^2)^2 + (g_{12} + g_{21})^2\right)} \end{array} \right\}. \tag{23}$$

Depending on the analysis frequency, Ω, the minimum and maximum tomographic signals correspond to one or the other sign in the previous expression.

One can verify that

$$\beta_{\rm opt}[\Omega_\pm] = \theta_\pm \tag{24}$$

which is an important result on which is based the principle of angular and spectral tomography. When realizing the angular tomography at an analysis frequency equal to the resonance frequency of dressed eigenmodes, the tomographic signals present a maximum along the eigenmode direction. This relation is general, in particular it also holds in the case of non-conservative force fields, even if the coupled eigenmodes orthogonality is broken in this case. This result is also independent of the damping rates that were here assumed equal. Note that this calculation is also valid in case of low quality factors, at the condition to realize the tomography at the mechanical resonance frequency, which does not always coincide with the frequency that maximizes displacement noise power. This observation anticipates the possibility to realize such measurements in air, even when the eigenmodes are not spectrally resolved.

In that situation, the maximum noise spectral density obtained at the eigenmode resonance can be written:

$$S_{\delta r_{\theta_\pm}}[\Omega_\pm] = \frac{S_F^{\rm th}}{M_{\rm eff}^2\Gamma^2\Omega_\pm^2}\left(1+\frac{(g_{12}-g_{21})^2}{(\Omega_+^2-\Omega_-^2)^2+\Gamma^2\Omega_\pm^2}\right) \tag{25}$$

where we have isolated the norm of the resonant mechanical susceptibility of the dressed oscillator $1/(-iM_{\rm eff}\Gamma\Omega_\pm)$, see later. The last fraction thus appear as an excess of noise, which only appears in a non-conservative force field and is proportional to $\mathrm{rot}_{\rm 2D}(F)^2$. We note that it can take large values, and this noise excess reaches $Q^2\mathrm{rot}_{\rm 2D}(F)^2/M_{\rm eff}^2\Omega_\pm^4$ at the bifurcation ($\Omega_-=\Omega_+$). The above expression is employed in Figure 2f of the manuscript to compute the expected noise spectral density deduced from the measured g_{ij} and to compare this value with the measured noise powers.

SUPPLEMENTARY NOTE 4 : Derivation of force field gradients from eigenfrequency shifts and eigenmode rotations

This section details how the four force gradient components g_{ij} can then be computed from the dressed modes rotation angles $\delta\theta_-\equiv\theta_--\theta_1$, $\delta\theta_+\equiv\theta_+-\theta_2$ and frequencies Ω_- and Ω_+.
The dressed modes frequencies are related to force gradients and cold frequencies by (Supplementary Equation 4):

$$\Omega_+^2+\Omega_-^2 \;=\; \Omega_{1||}^2+\Omega_{2||}^2 \tag{26}$$

$$(\Omega_+^2-\Omega_-^2)^2 \;=\; (\Omega_{2||}^2-\Omega_{1||}^2)^2+4g_{12}g_{21}. \tag{27}$$

The dressed modes orientations are:

$$\tan\delta\theta_- = \frac{g_{12}}{\Omega_{1||}^2-\Omega_-^2},\quad \tan\delta\theta_+ = \frac{g_{21}}{\Omega_+^2-\Omega_{2||}^2}. \tag{28}$$

We introduce $\mu\equiv\tan\delta\theta_-\tan\delta\theta_+$ and $\Delta\Omega_{||}^2=\Omega_{2||}^2-\Omega_{1||}^2$. Using the product of the above set of equations, we have:

$$4g_{12}g_{21} = \mu\Big(\Delta\Omega_{||}^2+\sqrt{\Delta\Omega_{||}^4+4g_{12}g_{21}}\Big)^2. \tag{29}$$

This expression of $4g_{12}g_{21}$ is injected into Supplementary Equation 27:

$$(\Omega_+^2-\Omega_-^2)^2 = \Delta\Omega_{||}^4+\mu\Big(\Delta\Omega_{||}^2+\sqrt{\Delta\Omega_{||}^4+4g_{12}g_{21}}\Big)^2 \tag{30}$$

which can be rewritten

$$(\Omega_+^2-\Omega_-^2)^2 = \Delta\Omega_{||}^4+\mu\Big(\Delta\Omega_{||}^2+\Omega_+^2-\Omega_-^2\Big)^2 \tag{31}$$

which is a second order equation in $\Delta\Omega_{||}^2$, yielding the two solutions: $\Delta\Omega_{||}^2 = (\Omega_+^2 - \Omega_-^2)\frac{-\mu\pm 1}{1+\mu}$. The solution involving the + sign is discarded because it imposes $g_{12}g_{21} = 0$ which is over-constraining. We thus have:

$$\Delta\Omega_{||}^2 = (\Omega_+^2 - \Omega_-^2)\frac{1-\mu}{1+\mu} \tag{32}$$

This equation permits to determine $\Delta\Omega_{||}^2 \equiv \Omega_2^2 - \Omega_1^2 + g_{11} - g_{22}$, and when combined with Supplementary Equation 26, one obtains the expression determining the parallel force field components:

$$g_{11} = \Omega_1^2 - \frac{1}{1+\mu}\left(\Omega_-^2 + \mu\Omega_+^2\right) \tag{33}$$

$$g_{22} = \Omega_2^2 - \frac{1}{1+\mu}\left(\Omega_+^2 + \mu\Omega_-^2\right) \tag{34}$$

The two shear force gradients are given by:

$$g_{12} = \frac{\tan\delta\theta_-}{2}\left(\Delta\Omega_{||}^2 + \Omega_+^2 - \Omega_-^2\right) \tag{35}$$

$$g_{21} = \frac{\tan\delta\theta_+}{2}\left(\Delta\Omega_{||}^2 + \Omega_+^2 - \Omega_-^2\right) \tag{36}$$

or

$$g_{12} = \frac{1}{1+\mu}(\Omega_+^2 - \Omega_-^2)\tan\delta\theta_- \tag{37}$$

$$g_{21} = \frac{1}{1+\mu}(\Omega_+^2 - \Omega_-^2)\tan\delta\theta_+ \tag{38}$$

We recall here that

$$\mu \equiv \tan\delta\theta_- \ \tan\delta\theta_+, \tag{39}$$

is a measured quantity.

SUPPLEMENTARY NOTE 5: Response measurements and tensorial susceptibility

Response measurements

Coherently driving the nanowire with a monochromatic external force of magnitude δF aligned along $\mathbf{e_F}$ generates a driven displacement $\boldsymbol{\delta}\mathbf{r}(t)$. In the stationary regime, when the transitory regime is finished, it can be written as $\boldsymbol{\delta}\mathbf{r}[\Omega] = \mathrm{Re}(\boldsymbol{\delta}\mathbf{r}[\Omega]e^{-i\Omega t}) = \mathrm{Re}(\boldsymbol{\chi}[\Omega]\delta F\mathbf{e}_\mathrm{F}e^{-i\Omega t})$. Due to the 2D and complex nature of the mechanical susceptibility, this corresponds to elliptical trajectories. We determine here the orientation and extension of the driven trajectories.

Experimentally, when realizing response measurements, we don't directly measure $\boldsymbol{\delta}\mathbf{r}[\Omega]$ but its projection along the measurements vectors $\delta r_{\ominus,\oplus}[\Omega]$ (forming an angle $\beta_{\ominus,\oplus}$ with the x axis) whose amplitude and phases are simultaneously acquired on a network analyzer featuring two synchronously read inputs. Since the measurements vectors are not always perpendicular, we first have to rebuild the projections of the driven trajectories in the (xz) cartesian basis, namely $\delta r_{x,z}[\Omega] \equiv \boldsymbol{\delta}\mathbf{r}[\Omega]\cdot\mathbf{e_{x,z}}$:

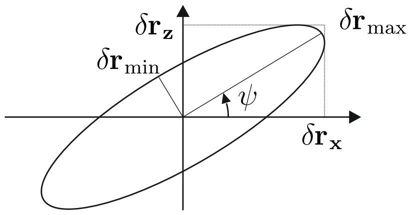

Supplementary Figure 8: **Parametrization of the driven trajectories.**

$$\begin{pmatrix} \delta r_x[\Omega] \\ \delta r_z[\Omega] \end{pmatrix} = \begin{pmatrix} \cos\beta_\ominus & \sin\beta_\ominus \\ \cos\beta_\oplus & \sin\beta_\oplus \end{pmatrix}^{-1} \cdot \begin{pmatrix} \delta r_\ominus[\Omega] \\ \delta r_\oplus[\Omega] \end{pmatrix} \tag{40}$$

The inverse matrix is always defined as long as the measurement angles are not parallel. The driven trajectories can be written for each frequency: $\boldsymbol{\delta}\mathbf{r}(t) = \mathrm{Re}\left((\delta r_x e^{i\varphi_x}\mathbf{e_x} + \delta r_z e^{i\varphi_z}\mathbf{e_z})e^{-i\Omega t}\right)$ where we have introduced the phases $\varphi_{x,z}[\Omega] \equiv \arg \delta r_{x,z}[\Omega]$. The phases involved here correspond to the dephasing of the measured projected displacement with respect to the driving force. Experimentally we have access to the dephasing between the input and outputs of the VNA, so that one has to take into account the detector response, the actuation delays to correctly establish it. Such trajectories are indeed ellipses with great/small radii $r_{\mathrm{max}}/r_{\mathrm{min}}$ forming an angle ψ with $\mathbf{e_x}$, see **Supplementary Figure 8**, given by:

$$r_{\mathrm{max}}^2 = \frac{\delta r_x^2 + \delta r_z^2}{2} + \frac{1}{2}\sqrt{(\delta r_x^2 - \delta r_z^2)^2 + 4\delta r_x^2 \delta r_z^2 \cos^2((\varphi_z - \varphi_x))} \tag{41}$$

$$r_{\mathrm{min}}^2 = \frac{\delta r_x^2 + \delta r_z^2}{2} - \frac{1}{2}\sqrt{(\delta r_x^2 - \delta r_z^2)^2 + 4\delta r_x^2 \delta r_z^2 \cos^2((\varphi_z - \varphi_x))} \tag{42}$$

$$\psi = \pi/2 + \frac{1}{2}\arctan\left(\frac{2\delta r_x \delta r_z \cos(\varphi_z - \varphi_x)}{\delta r_x^2 - \delta r_z^2}\right) \tag{43}$$

These expressions are used to plot the elliptical trajectories derived from the experimental response measurements all along the manuscript.

Tensorial susceptibility

In our experiment, we can in principle drive and measure the driven response of the nanowire along any readout and drive orientations, which allows to access to the tensorial character of the 2D susceptibility:

$$\chi_{\beta\phi}[\Omega] = \mathbf{e}_\beta \cdot \bar{\chi}[\Omega] \cdot \mathbf{e}_\phi \tag{44}$$

measured when one drives along $\mathbf{e}_\phi$ and measures along $\mathbf{e}_\beta$.

The susceptibility matrix is connected to its diagonal form by:

$$\bar{\chi}[\Omega] = \mathbf{R} \cdot \bar{\chi}_{\mathrm{diag}}[\Omega] \cdot \mathbf{R}^{-1} \tag{45}$$

where the diagonal matrix is

$$\bar{\chi}_{\mathrm{diag}}[\Omega] \equiv \begin{pmatrix} \chi_-[\Omega] & 0 \\ 0 & \chi_+[\Omega] \end{pmatrix} \tag{46}$$

with the eigen-susceptibilities $\chi_\pm[\Omega]$ given by:

$$\chi_\pm[\Omega] = \frac{1}{M_{\mathrm{eff}}\left(\Omega_\pm^2 - \Omega^2 - i\Omega\Gamma\right)} \tag{47}$$

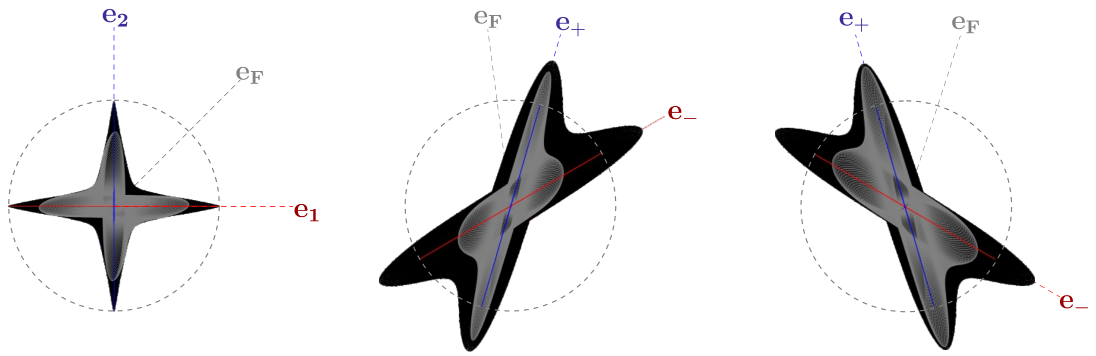

Supplementary Figure 9: **Set of driven trajectories in conservative and non conservative cases** Each graph displays a 2D representation of the driven trajectories of the NW using identical driving amplitudes but varying drive orientations. The driven trajectories corresponding to one particular drive angle labeled $\mathbf{e_F}$ are shown in gray. Depending on the drive frequency, the driven eclipses rotate in the 2D plane. On the contrary, the driven trajectories obtained when driving the NW along its eigenmode orientation are all aligned along the eigenmode direction. The are shown in red/blue for the +/- eigenmodes, the ellipses obtained at different drive frequencies are all aligned on top of each other. In black are shown the entire set of driven trajectories while scanning both the drive frequency and the drive orientation, sampling the entire 2π range of possible drive vector orientations. Left graph is obtained in absence of external force fell. The very same structure is obtained in presence of a conservative force field, rotated with the eigenmodes. The center graph corresponds to a non-conservative situation ($g_{ij} \propto (0, 2, -1, 0)$). The right graph corresponds to a non-conservative situation ($g_{ij} \propto (0, -2, 1, 0)$) . One can notice that in both non conservative cases, the maximum displacement measured along the eigenmode orientations is not obtained with a driving vector aligned along the eigenmode orientations (red/blue sets of trajectories).

which are equivalent to the one of a single 1D oscillator with a simple redefinition of the eigenfrequencies, the damping rates and effective being unchanged compared to the uncoupled situation. The transfer matrix $\mathbf{R}$, which permits to go from the initial $\mathbf{e_{1,2}}$ basis to the dressed $\mathbf{e}_{-,+}$ basis, can be chosen as:

$$\mathbf{R} = \begin{pmatrix} \Delta\Omega_\perp^2 & -g_{21} \\ g_{12} & \Delta\Omega_\perp^2 \end{pmatrix} \tag{48}$$

in which each column is proportional to the dressed eigenvectors expressed in the uncoupled basis. We point out that it does not depend on frequency. In the conservative case, it is proportional to a simple rotation matrix. We have:

$$\mathbf{R}^{-1} = \frac{1}{\Delta\Omega_\perp^{2\ 2} + g_{12}g_{21}} \begin{pmatrix} \Delta\Omega_\perp^2 & g_{21} \\ -g_{12} & \Delta\Omega_\perp^2 \end{pmatrix}. \tag{49}$$

The displacement-force relation can be expressed as :

$$\boldsymbol{\delta}\mathbf{r}[\Omega] = \mathbf{R} \cdot \bar{\chi}_{\mathrm{diag}}[\Omega] \cdot \mathbf{R}^{-1} \cdot \boldsymbol{\delta}\mathbf{F}[\Omega]. \tag{50}$$

Axial contributions

One can verify that the inverse transfer matrix $\mathbf{R}^{-1}$ reciprocally projects $\mathbf{e}_-$ on $\mathbf{e_1}$ so that if the driving force $\boldsymbol{\delta}\mathbf{F}[\Omega] = \delta F \mathbf{e_F}$ is oriented along the slow eigenmode $\mathbf{e}_-$, it generates as expected a displacement along the $\mathbf{e}_-$ axis (using that if $\mathbf{R}^{-1} \cdot \mathbf{e}_- = \lambda\, \mathbf{e_1}$, we also have: $\mathbf{R} \cdot \mathbf{e_1} = 1/\lambda\, \mathbf{e}_-$). As such, the driven displacement generated when driving along $\mathbf{e}_-$ is

$$\boldsymbol{\delta}\mathbf{r}[\Omega] = \mathbf{R} \cdot \bar{\chi}_{\mathrm{diag}}[\Omega] \cdot \mathbf{R}^{-1} \cdot \mathbf{e}_- \delta F = \chi_-[\Omega] \delta F \mathbf{e}_-. \tag{51}$$

This expression which simply reformulates the diagonalization of the susceptibility matrix, suggests however that the mechanical susceptibility evaluated when driving along the dressed eigenmode orientations remains uniaxial (as

expected for an eigenmode) and preserves a standard monodimensional form, where only the mechanical resonance frequency is modified:

$$\chi_{\pm\pm}[\Omega] \equiv \mathbf{e}_\pm \cdot \bar{\chi}[\Omega] \cdot \mathbf{e}_\pm = \chi_\pm[\Omega] \tag{52}$$

From this remark we can also compute what the fluctuation dissipation theorem would predict to measure when orienting the readout vector along one of the dressed eigenmode orientation ($\mathbf{e}_\beta = \mathbf{e}_-$). The expected measured noise spectral density is given by:

$$S_{\delta r_\pm}^{\mathrm{FDT}}[\Omega] = \frac{2k_BT}{|\Omega|}\,|\mathrm{Im}\chi_{\pm\pm}[\Omega]| = \frac{2\Gamma k_BT}{M((\Omega_\pm^2-\Omega^2)^2+\Omega^2\Gamma^2)} \tag{53}$$

which is similar to the standard 1D expression for a thermal noise.
Instead, a noise increase by a factor of 40 is observed in the experiment when probing at eigenmode resonance frequencies ($S_{\delta r_\pm}[\Omega_\pm]$), providing a first hint towards a violation of the FDT theorem, simply based on the discrepancy observed in the measured noise magnitude at resonance. The observed excess of noise (shown in Fig. 1e of the manuscript and analyzed in Fig. 2f where $S_{\delta r_-}[\Omega_\pm]$ is shown) cannot thus be attributed to a divergence of the NW axial susceptibility, but originates instead from the breaking of eigenmodes orthogonality. This point will now be clarified by inspecting the non-axial contribution to the tensorial susceptibility.

Non-axial contributions

We now investigate the non-axial contributions to the tensorial susceptibility. For simplicity, we continue to investigate the displacement measured along one eigenmode orientation, but now give the freedom to the driving force vector to be oriented in an arbitrary direction $\mathbf{e_F}$, forming an angle θ_F with $\mathbf{e_1}$.

It is first interesting to plot the ensemble of responses obtained by scanning the drive force orientation $\mathbf{e_F}$ all around the horizontal plane while maintaining a fixed drive strength, by sweeping the drive frequency across both eigenmodes and plotting the steady state trajectories in 2D. The result is shown in **Supplementary Figure 9** both in the conservative (left) and non-conservative (center, right) cases. This ensemble of responses is compared to the response measured when driving the nanowire along the eigenmodes orientations (red/blue curves, the maximum magnitude is highlighted by the dashed circle). We note that in the non-conservative cases the largest driven displacement along the eigenmode orientations is not obtained by choosing a drive vector oriented along the eigenmode itself. For example, the set of gray curves are obtained for the drive vector shown in gray, which is not aligned with the eigenmode orientations, but produces driven displacements going significantly beyond the maximum displacement measured in the aligned situation (red/blue curves). We insist in mentioning that the drive strength is unchanged for all driven responses shown in this images. This rather surprising observation is at the heart of the measured noise increase- and thus of the violation of the fluctuation dissipation theorem- in our system.
We now evaluate the non-axial terms of the mechanical susceptibility at the resonance of the slow eigenmode to investigate the situation where the drive vector is not oriented along the eigenmode:

$$\chi_{-\,F}[\Omega_-] \;\equiv\; \mathbf{e}_- \cdot \bar{\chi}[\Omega_-] \cdot \mathbf{e_F} = \mathbf{e}_- \cdot \boldsymbol{R} \cdot \bar{\chi}_{\mathrm{diag}}[\Omega_-] \cdot \boldsymbol{R}^{-1} \cdot \mathbf{e_F} \tag{54}$$

At that step, we can assume that $\Omega_+ - \Omega_- > \Gamma$. This criteria is less stringent than the criteria where eigenmode are resolved in individual thermal noise measurements and can still apply close to the bifurcation (although the excess of noise and its peculiar spectral dependence may prevent from resolving both peaks on the experimental spectra). In that situation we can assume that $|\chi_-[\Omega_-]| \gg |\chi_+[\Omega_-]|$ and reduce the above equation to its dominant contribution:

$$\chi_{-\,F}[\Omega_-] \approx \chi_-[\Omega_-]\frac{1}{{\Delta\Omega_\perp^2}^2 + g_{12}g_{21}}\left(\Delta\Omega_\perp^2\cos\theta_F + g_{21}\sin\theta_F\right) \tag{55}$$

The last parenthesis is maximal with respect to θ_F when $\theta_F = \theta_{-F}^{\mathrm{opt}}$ defined by:

$$\tan\theta_{-F}^{\mathrm{opt}} = \frac{g_{21}}{\Delta\Omega_\perp^2} = \frac{-1}{\tan\theta_+} = \tan(\theta_+ + \pi/2). \tag{56}$$

where we have used Supplementary Equation (7). As such we have shown that, modulo π:

$$\theta^{\rm opt}_{\mp F} = \theta_\pm + \pi/2 \tag{57}$$

which is an important expression to understand the observed excess of noise.
First in a conservative force field, when eigenmodes preserve their orthogonality, this expression is intuitive: to maximize the displacement along one eigenmode orientation, it is necessary to drive the system along the eigenmode orientation, which means perpendicularly to the other eigenmode.
In the non-conservative case, the consequence is rather surprising: to obtain the largest displacement along one eigenmode orientation, it is more efficient to drive the NW along a direction which is not the eigenmode direction itself, and more precisely, the optimum direction is perpendicular to the other eigenmode orientation. A direct signature of this effect is visible in the experimental data shown in Fig. 2a, 4 of the manuscript, where the responses are all taken with the same driving strength, but a significantly larger oscillation amplitude is observed in the data taken in a large non-conservative force field, despite the fact that the force was not aligned with the eigenmodes but instead, almost perpendicular to the eigenmodes which converge towards a fixed orientation when approaching the bifurcation.
A numerical representation of the effect is shown in **Supplementary Figure 10** where the dependence of $\chi_{\pm\,F}[\Omega_\pm]$ on the drive orientation $\mathbf{e_F}$ is shown.
The remark permits to come to a simple geometrical interpretation of the excess of noise observed experimentally (Fig. 2f). Our description in term of 2 independent Langevin forces $\delta F_{1,2}$ acting along $\mathbf{e_{1,2}}$ can also be realized with any another set of independent Langevin forces acting along 2 other perpendicular orientations, for example $\mathbf{e}_-$ and its perpendicular: $\mathbf{e_y}\wedge\mathbf{e}_-$. In that situation the second Langevin force, which does not play any role in the noise measured along the eigenmode orientation in a conservative force field, will start to have an increasing contribution with the breaking of the eigenmode orthogonality, which leads to a non-zero non- axial susceptibility. As a consequence, the noise measured along one orientation is subject to cross contributions from each orthogonal Langevin force to the total measured noise power. Since the axial contribution has a comparable magnitude as in absence of force field, the role of the non axial contribution is to increase the measured noise power, explaining the observed excess of thermal noise.

Geometrical interpretation

Having inspected the properties of the axial and transverse components of the susceptibility, we will now demonstrate that the measured spectrum takes a very simple form when making those 2 contributions appear.
The measured projected signal can be written:

$$\begin{aligned}\delta r_\beta[\Omega] &= \mathbf{e}_\beta\cdot\bar{\chi}\cdot(\delta F_1\mathbf{e_1}+\delta F_2\mathbf{e_2})\\ &= \mathbf{e}_\beta\cdot\bar{\chi}\cdot\left(\delta F_\beta^{||}\mathbf{e}_\beta+\delta F_\beta^{\perp}\mathbf{e}_\beta^{\perp}\right)\\ &= \chi_\beta^{||}[\Omega]\delta F_\beta^{||}+\chi_\beta^{\perp}[\Omega]\delta F_\beta^{\perp}\end{aligned} \tag{58}$$

where we have introduced the unitary vector $\mathbf{e}_\beta^\perp$ perpendicular to the measurement vector $\mathbf{e}_\beta$ so that the base $(\mathbf{e}_\beta, \mathbf{e}_\beta^\perp)$ is direct, the axial and transverse susceptibilities:

$$\chi_\beta^{||}[\Omega]\equiv\mathbf{e}_\beta\cdot\bar{\chi}\cdot\mathbf{e}_\beta \ \text{ and } \ \chi_\beta^{\perp}[\Omega]\equiv\mathbf{e}_\beta\cdot\bar{\chi}\cdot\mathbf{e}_\beta^{\perp} \tag{59}$$

and a new set of Langevin forces $(\delta F_\beta^{||}, \delta F_\beta^\perp)$ defined as:

$$\begin{pmatrix}\delta F_\beta^{||}\\ \delta F_\beta^{\perp}\end{pmatrix} = \begin{pmatrix}\cos\beta & \sin\beta\\ -\sin\beta & \cos\beta\end{pmatrix}\cdot\begin{pmatrix}\delta F_1\\ \delta F_2\end{pmatrix}. \tag{60}$$

They present the same spectral density as the original Langevin forces and no cross-correlation: $\langle\delta F_\beta^{||}(t)\delta F_\beta^{\perp}(t')\rangle = 0$. One can then compute the measured noise spectral density as:

$$S_{\delta r_\beta}[\Omega] = S_F^{\rm th}\left(|\chi_\beta^{||}[\Omega]|^2+|\chi_\beta^{\perp}[\Omega]|^2\right). \tag{61}$$

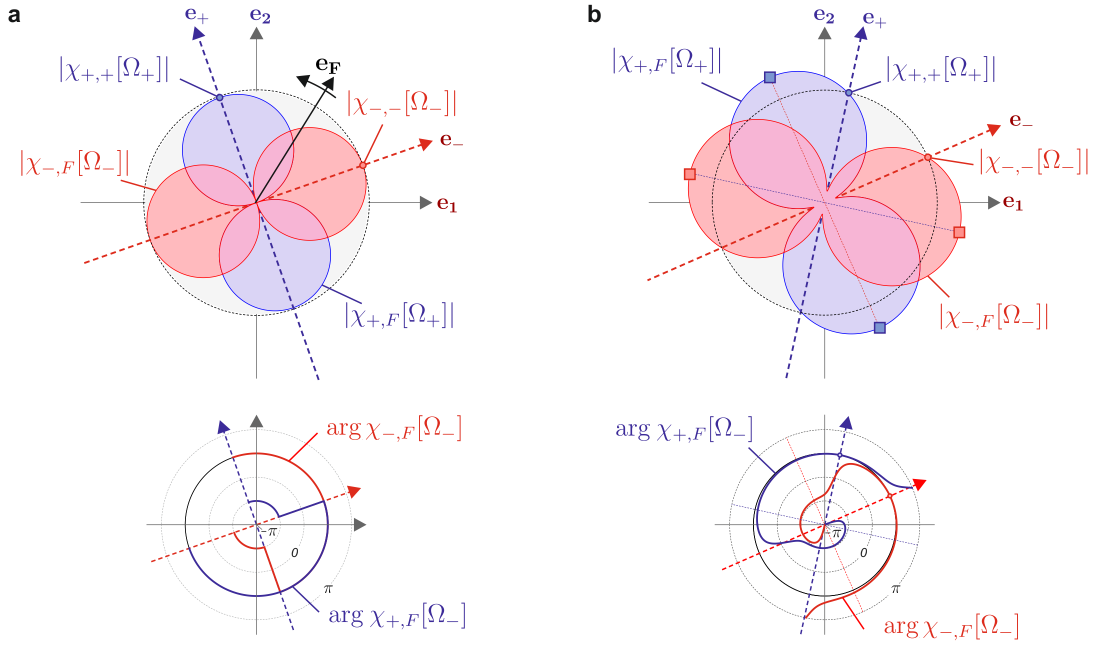

Supplementary Figure 10: **Influence of drive angle on the tensorial susceptibility** Illustration of the dependence on the drive vector orientation of the cross susceptibilities $\chi_{\pm F}[\Omega_\pm]$ in the cases of a conservative (**a**) and non conservative (**b**) force fields. The amplitude and the phase of the crossed susceptibilities are shown in the upper and lower panels. In the conservative situation, the behavior is rather intuitive: the maximum displacement measured along one eigenmode at resonance is obtained by driving the system along its eigenmode. The tensorial susceptibility engaged is then $\chi_{--}[\Omega_-] = \frac{i}{M_{\rm eff}\Omega_-\Gamma}$ for example at the point marked by a red point. The situation is very different in a non conservative force field: (right panels). When driving the nanowire at resonance of mode $-$ for example, it is possible to generate driven displacements measured along the eigenmode orientation $\mathbf{e}_-$ larger than the ones obtained by aligning the force vector along the eigenmode, by choosing a different drive vector orientation. In particular, the maximum displacement is obtained by choosing a drive orientation exactly perpendicular to the other eigenmode orientation (orientations marked by squares). This is a direct consequence of the coupled dynamics of the eigenmodes and represents a geometric explanation of the origin of the violation of the fluctuation dissipation theorem since in that case the second Langevin force is now capable of driving very efficiently the eigenmode under investigation.

This reasoning can be used to compute the projected displacement measured along the eigenvector orientation $\mathbf{e}_-$. To do so we refer to the axial and transverse susceptibilities introduced above. We have already seen that:

$$\chi_-^{\parallel}[\Omega] \equiv \mathbf{e}_- \cdot \bar{\chi}[\Omega] \cdot \mathbf{e}_- = \frac{1}{M_{\rm eff}(\Omega_-^2 - \Omega^2 - i\Omega\Gamma)} \tag{62}$$

and we can verify, using the diagonalized form of the susceptibility given in Supplementary Equation (45) and the fact that $\Delta\Omega_\perp^2(\Omega_+^2 - \Omega_-^2) = \Delta\Omega_\perp^{2\;2} + g_{12}g_{21}$ that:

$$\chi_-^{\perp}[\Omega] \equiv \mathbf{e}_- \cdot \bar{\chi}[\Omega] \cdot \mathbf{e}_-^{\perp} = M_{\rm eff}\,(g_{12} - g_{21})\,\chi_-^{\parallel}[\Omega]\chi_+^{\parallel}[\Omega] \tag{63}$$

so that the noise spectrum measured along the eigenmode orientation takes the simple form:

$$S_{\delta r_-}[\Omega]/S_F^{\rm th} = |\chi_-^{\parallel}[\Omega]|^2\left(1 + (g_{12} - g_{21})^2 M_{\rm eff}^2 |\chi_+^{\parallel}[\Omega]|^2\right). \tag{64}$$

so that:

$$S_{\delta r_-}[\Omega] = \frac{2\Gamma k_B T}{M_{\rm eff}((\Omega_-^2 - \Omega^2)^2 + \Omega^2\Gamma^2)}\left(1 + \frac{(g_{12} - g_{21})^2}{((\Omega_+^2 - \Omega^2)^2 + \Omega^2\Gamma^2)}\right). \tag{65}$$

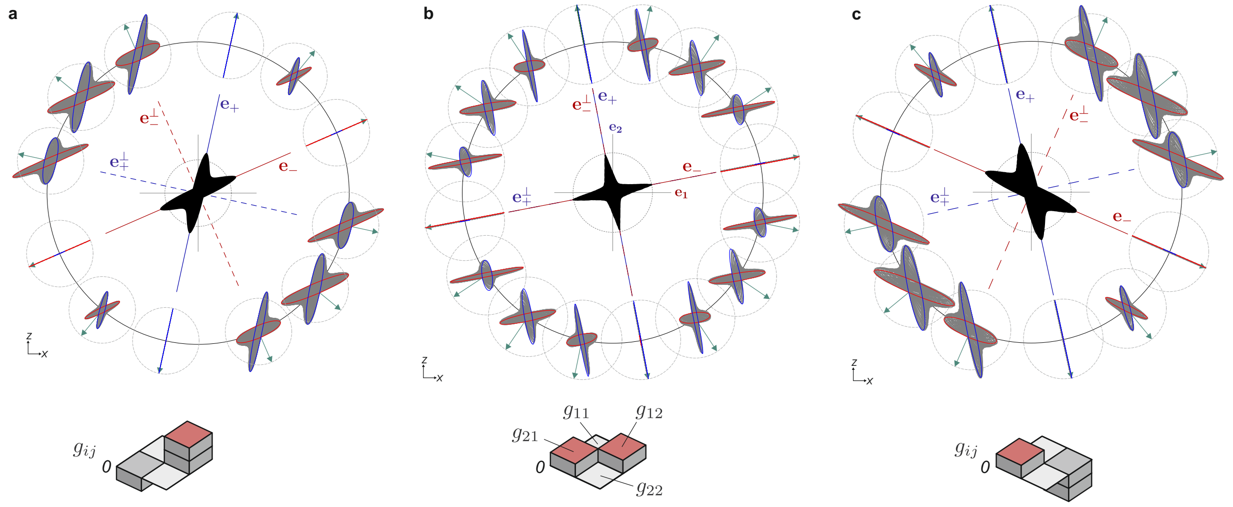

Supplementary Figure 11: **Tensorial susceptibility** Each graph displays a 2D representation of the driven trajectories of the NW using identical driving amplitudes but varying drive orientations in 3 different force field gradients, whose relative magnitude is sketched in the lower representation. The left, center and right panels feature positive, null and negative vorticity. In each plot the central image represents the entire set of driven trajectories that can be achieved for all drive orientations and all drive frequencies. The surrounding plots represents the set of driven trajectories achievable for all drive frequencies for one single force orientation indicated as a dark green arrow. The dressed eigenmodes'orientations are shown in the center, as well as orthogonal orientations $\mathbf{e}_{\pm}^{\perp}$. When the force vector is aligned with the eigenmode, a uniaxial response is observed, as expected. When it is not aligned, the driven response can explore both directions in space. In the conservative situation, the maximum driven amplitude is maximal in the uniaxial situation. On the contrary, in the non-conservative situation, larger displacements can be achieved under transverse actuation, with a maximum magnitude when $\mathbf{e_F} = \mathbf{e}_{\pm}^{\perp}$.

The above formula can be evaluated at the eigenmode frequency to obtain tomographic expression of $S_{\delta r_-}[\Omega_-]$ given in the manuscript:

$$S_{\delta r_-}[\Omega] = \frac{2k_B T}{M_{\text{eff}}\Omega_-^2\Gamma}\left(1 + \frac{(\text{rot}\mathbf{F})^2}{M_{\text{eff}}^2((\Omega_+^2 - \Omega_-^2)^2 + \Omega_-^2\Gamma^2)}\right). \tag{66}$$

The 2 contributions in the parenthesis are now very clear, the first term represents the uniaxial response of the eigenmode, which does not present any noise increase at resonance compared to the uncoupled case. The second term represents the transverse contribution, where the system is driven by the second independent Langevin force vector, $\delta F_-^{\perp}$, acting perpendicularly to the eigenmode orientation. This last term only contributes in the non-conservative case. Due to the increased response observed along transverse directions, it significantly contributes when the eigenmode orientations become non-perpendicular. When approaching the bifurcation, both eigenmode orientations converge towards the same direction, this also means that $\mathbf{e}_-^{\perp} \to \mathbf{e}_+^{\perp}$, so that the transverse susceptibility reaches its maximal contribution as studied above.

SUPPLEMENTARY NOTE 6: Fluctuation dissipation relation

We present here a 2D formulation of the excess of noise and connect it to the non-axial contributions in the mechanical susceptibility.

The displacement thermal noise projected along $\mathbf{e}_\beta \equiv \begin{pmatrix} \cos\beta \\ \sin\beta \end{pmatrix}$ and $\mathbf{e}_\beta^{\perp} \equiv \begin{pmatrix} -\sin\beta \\ \cos\beta \end{pmatrix}$ reads:

$$\delta r_\beta = \delta F_1 \left(\cos\beta\, \chi_{11} + \sin\beta\, \chi_{21}\right) + \delta F_2 \left(\cos\beta\, \chi_{12} + \sin\beta\, \chi_{22}\right) \tag{67}$$

$$\delta r_{\beta^\perp} = \delta F_1 \left(-\sin\beta\, \chi_{11} + \cos\beta\, \chi_{21}\right) + \delta F_2 \left(-\sin\beta\, \chi_{12} + \cos\beta\, \chi_{22}\right) \tag{68}$$

we also have the longitudinal susceptibilities

$$\chi_{\beta\beta} = \cos^2\beta\,\chi_{11} + \sin^2\beta\,\chi_{22} + \cos\beta\sin\beta\,(\chi_{12}+\chi_{21}) \quad (69)$$
$$\chi_{\beta^\perp\beta^\perp} = \sin^2\beta\,\chi_{11} + \cos^2\beta\,\chi_{22} - \cos\beta\sin\beta\,(\chi_{12}+\chi_{21}) \quad (70)$$

and the transverse components:

$$\chi_{\beta\beta^\perp} = \cos^2\beta\,\chi_{12} - \sin^2\beta\,\chi_{21} + \cos\beta\sin\beta\,(\chi_{22}-\chi_{11}) \quad (71)$$
$$\chi_{\beta^\perp\beta^{\perp\perp}} = \sin^2\beta\,\chi_{12} - \cos^2\beta\,\chi_{21} - \cos\beta\sin\beta\,(\chi_{22}-\chi_{11}) \quad (72)$$

where $\mathbf{e}_\beta^{\perp\perp} \equiv -\mathbf{e}_\beta$ for preserving the $+\pi/2$ orientation in the definition of transverse susceptibilities.

One can show that:

$$\sum_{\mu=\beta,\beta^\perp} S_{\delta r_\mu}[\Omega] = S_F^{\mathrm{th}}\left(|\chi_{\beta\beta}|^2 + |\chi_{\beta\beta^\perp}|^2 + |\chi_{\beta^\perp\beta^{\perp\perp}}|^2 + |\chi_{\beta\beta}|^2\right) \quad (73)$$
$$= S_F^{\mathrm{th}}\left(|\chi_{11}|^2 + |\chi_{12}|^2 + |\chi_{21}|^2 + |\chi_{22}|^2\right) \quad (74)$$
$$(75)$$

so that the sum is independent of the choice of perpendicular measurement vectors $(\beta,\beta^\perp)$ employed. Then one can also show that:

$$\sum_{\mu=\beta,\beta^\perp} \frac{2k_BT}{|\Omega|}|\mathrm{Im}\chi_{\mu\mu}[\Omega]| = \sum_{\mu=1,2} \frac{2k_BT}{|\Omega|}|\mathrm{Im}\chi_{\mu\mu}[\Omega]| \quad (76)$$
$$= S_F^{\mathrm{th}}\left(|\chi_{11}|^2 + |\chi_{22}|^2 + 2\mathrm{Re}(\chi_{12}^\star\chi_{21})\right). \quad (77)$$

To demonstrate the second relation, it is useful to note that $\frac{2k_BT}{\Omega}\mathrm{Im}\chi_{ii} = S_F^{\mathrm{th}}(|\chi_{ii}|^2 + \mathrm{Re}(\chi_{12}^\star\chi_{21}))$, which is positive in our case. Finally, subtracting the 2 equations above, one arrives at the result that:

$$\sum_{\mu=\beta,\beta^\perp}\left(S_{\delta r_\mu}[\Omega] - \frac{2k_BT}{|\Omega|}\,|\mathrm{Im}\chi_{\mu\mu}[\Omega]|\right) = S_F^{\mathrm{th}}\,|\chi_{12}-\chi_{21}|^2 \quad (78)$$

This expression is directly a patch of the fluctuation-dissipation theorem in our case. The argument in the parenthesis is directly the standard uniaxial formulation of the FDT theorem. It weighted spectral integral is also the one that appear in the Harada-Sasa theorem, which is proportional to the work of the non-conservative forces, see below. Here we give a direct interpretation of this deviation which is directly connected to the susceptibility matrix asymmetry, caused by the non-reciprocal coupling. We also have:

$$\sum_{\mu=\beta,\beta^\perp}\left(S_{\delta r_\mu}[\Omega] - \frac{2k_BT}{|\Omega|}|\mathrm{Im}\chi_{\mu\mu}[\Omega]|\right) = \frac{2k_BT}{M}\frac{(g_{12}-g_{21})^2}{\left((\Omega_+^2-\Omega^2)^2+\Omega^2\Gamma^2\right)\left((\Omega_-^2-\Omega^2)^2+\Omega^2\Gamma^2\right)} \quad (79)$$

From this expression we understand that the noise excess dominantly appears close to the dressed eigenfrequencies, as observed experimentally. We can also write a generic formulation:

$$\sum_{\mu=\beta,\beta^\perp}\left(S_{\delta r_\mu}[\Omega] - \frac{2k_BT}{|\Omega|}|\mathrm{Im}\chi_{\mu\mu}[\Omega]|\right) = \frac{2k_BT}{|\Omega|}|\mathrm{Im}\left\{\boldsymbol{\chi}^{-1}\right\}_{ii}|\,\left|\chi_-^\perp[\Omega]|\chi_+^\perp[\Omega]\right| \quad (80)$$

valid for isotropically damped 2D nanomechanical systems, including over- and under-damped optical tweezers experiments.

SUPPLEMENTARY NOTE 7: Signal Processing and Analysis

We expose here the procedure employed to analyse the experimental data on thermal noise and response measurements.

Determination of the measurement vectors ($\mathbf{e}_{\pm}$).

Prior to each measurement, the measurement vectors are determined. To do so the experiment is piezo displaced as a whole (NW and green objective) with respect to the readout laser beam in order to evaluate the local variations of the reflected intensity maps ($V_{\ominus,\oplus}(\mathbf{r})$). The routine acquires the reflected intensities in 25 positions on a 20 nm XZ grid around the measurement position ($\mathbf{r_0}$) and the tangent plane is subsequently calculated allowing to compute $\boldsymbol{\nabla} V_{\ominus,\oplus}|_{\mathbf{r_0}}$, as well as higher order derivatives, which permits to estimate the linear readout range. The normalized measurement vectors are subsequently reconstructed: $\mathbf{e}_{\ominus,\oplus} = \boldsymbol{\nabla} V_{\ominus,\oplus}|_{\mathbf{r_0}} / |\, \boldsymbol{\nabla} V_{\ominus,\oplus}|_{\mathbf{r_0}} \,|$

HF/DC conversion efficiency.

The reflected images are obtained from the low frequency outputs of the photodiode amplifier while the dynamical vibration signals are encoded on their HF outputs (cutoff around 1 kHz). The detector response has been carefully calibrated in order to determine the exact conversion gain of each photodiode quadrant in each output (sum and difference) for both HF and DC channels. These calibrations are realized using a known intensity modulation produced by the AOM (swept across the desired measurement frequency span), and both the gain and the phase delays are recorded for each channel.

Reconstruction of the projected displacements.

The NW oscillations are encoded on the output channels as voltage fluctuations : $\delta V_{\ominus,\oplus}(t)$ and converted into projective displacements using the measurement vectors $\boldsymbol{\nabla} V_{\ominus,\oplus}|_{\mathbf{r_0}}$ determined above and the HF/DC conversion factors $G_{\ominus,\oplus}$:

$$\delta r_{\ominus,\oplus} = \delta V_{\ominus,\oplus} / |\, \boldsymbol{\nabla} V_{\ominus,\oplus}|_{\mathbf{r_0}} \,| / G_{\ominus,\oplus}. \tag{81}$$

This procedure is identically applied when recording noise spectra or response measurements on spectrum and network analyzers.

Determination of the uncoupled mechanical properties from thermal noise spectra.

They are determined in absence of green pump light, by simultaneously adjusting the thermal noise spectra recorded on each measurement channel: $S_{\delta r_{\ominus,\oplus}}[\Omega]$ using Supplementary Equation 18 with $g_{ij} = 0$ and $\mathbf{e}_\beta = \mathbf{e}_{\ominus,\oplus}$. This allows to determine the uncoupled frequencies $\Omega_{1,2}$, damping rate Γ and effective mass M_{eff} taken identical for both modes. Measurement at several readout optical powers also permit to verify that the NW is thermalized at room temperature T and that the probe beam has a negligible impact on the NW dynamics.

Determination of the uncoupled mechanical properties from response measurements.

To do so we use the optical drive exerted by the green power, (piezo actuation is not spectrally flat so it is not employed for advanced measurements): a weak green optical power is applied (2 μW average), and intensity modulated by the acousto-optic modulator (10 % modulation depth). The modulation frequency $\Omega_{\mathrm{mod}}/2\pi$ is swept across both eigenmodes and the nanowire response is simultaneously acquired on both measurement channels of a VNA, see Fig. 3e for example. For a given driving frequency, the driven displacement measured on each channel is given by $\delta r_{\ominus,\oplus}(t) = \mathrm{Re}\left(\delta r_{\ominus,\oplus}[\Omega_{\mathrm{mod}}] e^{-i\Omega_{\mathrm{mod}} t}\right)$ where:

$$\delta r_{\ominus,\oplus}[\Omega_{\mathrm{mod}}] = \mathbf{e}_{\ominus,\oplus} \cdot \bar{\chi}[\Omega_{\mathrm{mod}}] \cdot \delta F \, \mathbf{e}_{\mathrm{F}}. \tag{82}$$

These expressions are evaluated using the uncoupled mechanical susceptibility $\bar{\chi}$ (see Supplementary Equation 2 with $g_{ij} = 0$) and employed to fit the experimental traces both in amplitude and in phase. Note that the dephasing of the optical drive force with respect to the modulation voltage sent by the VNA must be independently measured. By doing so, the optical force can be verified to be instantaneous and to have a flat response in the frequency span considered here. For the fitting, the free parameters are $\Omega_{1,2}$, $\Gamma, M, \mathbf{e_{1,2}}$ and the force magnitude δF and orientation $\mathbf{e_F}$. Once the response measurements are realized, the NW properties can be compared to the results obtained from

thermal noise analysis. The response measurements can also be represented in the 2D plane by producing an ellipse for each drive frequency, as explained above, on top of which can be added the measurement vectors, uncoupled eigenmodes orientations and drive orientation (see Fig. 1c).

Determination of force field gradients (g_{ij}) from thermal noise spectra.

We record the thermal noise spectra in presence of the external coupling force field (green power turned on). Both projected spectra are recorded and simultaneously adjusted using Supplementary Equation 18 with the uncoupled mechanical parameters ($\Omega_{1,2}, \mathbf{e_{1,2}}, \Gamma, M, T$) and measurement vectors being fixed to their above determined values. Here, the only free parameters in the fit are the 4 force field gradients g_{ij}. A flat detection background spectrum is also added to perfectly fit the experimental traces (located at -30 dB below the peaks level). Once the force field gradients are determined, the local linearized force field map can be established by plotting the vector flow $\mathbf{f}(\delta\mathbf{r}) = \bar{g} \cdot \boldsymbol{\delta}\mathbf{r}$ as shown in Fig. 3adg for example.

Fitting of the response measurements in presence of an external force field.

Response measurements in presence of the external force field are acquired on the VNA as explained above. The response measured on each channel are fitted using the same procedure as above, except that now we use the fully dressed 2D mechanical susceptibility of Supplementary Equation 2. Depending on the purpose, one can choose to let the force field gradients g_{ij} as free fitting parameters, or to use the value determined from thermal noise spectra measurements. In that situation, which is the one in which the response measurements are analyzed in the FDR section (Fig. 3), the only fitting parameters are the force magnitude and orientation δF and $\mathbf{e_F}$.

SUPPLEMENTARY NOTE 8: Thermodynamical considerations

Entropy production

It was shown, for example by Zamponi and collaborators [1], that the motion in a non-conservative force is accompanied by a net entropy production rate. We now calculate the total produced entropy by very slightly adapting the derivation of [1] (the only adaptations being the introduction of the restoring term in the equation of motion, the hypotheses of a memory-less and delta-correlated bath.

The occurrence probability of a segment of trajectory $\delta\mathbf{r}(t)$ defined for $t \in [-\tau/2, \tau/2]$ for a certain solution of the Fokker-Planck equation $\mathcal{P}$ (here, we will always take the steady-state probability distribution) is denoted by $\mathcal{P}\{\delta\mathbf{r}\}$. Kurchan [2] and Lebowitz and Spohn [3] showed that the entropy produced during the time τ could be written, up to some boundary terms, as a function of the imbalance between the probabilities of the trajectory $\delta\mathbf{r}$ and of its time-reversed counterpart $\delta\tilde{\mathbf{r}}$:

$$S_\tau = k_B \ln\left(\frac{\mathcal{P}\{\delta\mathbf{r}\}}{\mathcal{P}\{\delta\tilde{\mathbf{r}}\}}\right). \tag{83}$$

Strictly speaking, this entropy has been identified by Seifert [4] to be the entropy increase in the medium surrounding the system, while the total entropy also includes the entropy of the system. However, according to this partition of the entropy, the entropy of the system precisely consists in the boundary terms which will here be removed, while the entropy given in Supplementary Equation 83 will be shown to diverge with time. Therefore we will follow [1] in identifying this entropy as the total entropy production during time τ.
The probability of a (2D) Langevin process over time τ is written [1, 5]:

$$\mathcal{P}\{\delta\mathbf{F}_{\text{th}}\} \propto \exp\left[-\frac{1}{2}\iint dt\, dt'\, \delta\mathbf{F}_{\text{th}}(t)\cdot\delta\mathbf{F}_{\text{th}}(t')\,\frac{\delta(t-t')}{2\Gamma k_B T M}\right] \tag{84}$$

where $\delta(t-t')$ is used here because of our assumption that the thermal bath is memoryless and delta-correlated.
Using the argument of [1], the integrals in this expression (and all following integrals) can simply be truncated to $t, t' \in [-\tau/2, \tau/2]$ up to some boundary terms as the processes rapidly decohere. Since there is a bijection between the segments of Langevin processes and the segments of trajectories, the probability $\mathcal{P}\{\delta\mathbf{r}\}$ can be deduced from this last

expression by replacing $\delta\mathbf{F}_{\rm th}(t)$ by its expression in terms of $\delta\mathbf{r}(t)$ and its time derivatives provided by the equation of motion:

$$\mathcal{P}\{\delta\mathbf{r}\} \propto \exp\left[-\frac{1}{2}\int\frac{dt}{2\Gamma k_B T M}\Big(M\delta\ddot{\mathbf{r}}(t)+M\Gamma\delta\dot{\mathbf{r}}(t)+M\underline{\underline{\Omega}}^2\delta\mathbf{r}(t)-\Big(\delta\mathbf{r}(t)\cdot\boldsymbol{\nabla}\Big)\mathbf{F}(\mathbf{r_0})\Big)^2\right], \tag{85}$$

where $\underline{\underline{\Omega}} = \begin{pmatrix}\Omega_1 & 0\\ 0 & \Omega_2\end{pmatrix}$ and $\mathbf{F}(\mathbf{r_0})$ is the external (here, optical) force field at the average nanowire position $\mathbf{r_0}$. The time reversal only affects the signs of the friction terms $M\Gamma\delta\dot{\mathbf{r}}(t)$:

$$\mathcal{P}\{\delta\tilde{\mathbf{r}}\} \propto \exp\left[-\frac{1}{2}\int\frac{dt}{2\Gamma k_B T M}\Big(M\delta\ddot{\mathbf{r}}(t)-M\Gamma\delta\dot{\mathbf{r}}(t)+M\underline{\underline{\Omega}}^2\delta\mathbf{r}(t)-\Big(\delta\mathbf{r}(t)\cdot\boldsymbol{\nabla}\Big)\mathbf{F}(\mathbf{r_0})\Big)^2\right], \tag{86}$$

We are then simply left with a series of entropy terms:

$$S_\tau = S_\tau^{(\rm kin)} + S_\tau^{(\rm rest)} + S_\tau^{(\rm F)} \tag{87}$$

with

$$S_\tau^{(\rm kin)} \equiv -\int_{-\tau/2}^{\tau/2} dt\,\frac{M}{T}\,\delta\dot{\mathbf{r}}(t)\delta\ddot{\mathbf{r}}(t) = -\frac{\Delta E_{\rm kin}}{T} \tag{88}$$

$$S_\tau^{(\rm rest)} \equiv -\int_{-\tau/2}^{\tau/2} dt\,\frac{M}{T}\,\delta\dot{\mathbf{r}}(t)\cdot\underline{\underline{\Omega}}^2\delta\mathbf{r}(t) = -\frac{\Delta E_{\rm p}^{(\rm rest)}}{T} \tag{89}$$

$$S_\tau^{(\rm F)} \equiv \int_{-\tau/2}^{\tau/2} dt\,\frac{1}{T}\,\delta\dot{\mathbf{r}}(t)\cdot\Big[\Big(\delta\mathbf{r}(t)\cdot\boldsymbol{\nabla}\Big)\mathbf{F}(\mathbf{r_0})\Big] = S_\tau^{(\rm F,c)} + S_\tau^{(\rm F,nc)} \tag{90}$$

where we identified the variation of the kinetic energy $\Delta E_{\rm kin}$ and of the restoring potential energy $\Delta E_{\rm p}^{(\rm rest)}$ in the interval τ. The last entropy term is due to the work of the external force and has to be decomposed to distinguish the contributions of the conservative and the non-conservative parts of the force:

$$S_\tau^{(\rm F,c)} \equiv \frac{M}{T}\left[\Big(g_{11}-\Omega_1^2\Big)\frac{\delta r_1^2}{2}+\Big(g_{22}-\Omega_2^2\Big)\frac{\delta r_2^2}{2}+\Big(\frac{g_{12}+g_{21}}{2}\Big)\delta r_1\delta r_2\right]_{-\tau/2}^{\tau/2} = -\frac{\Delta E_p^{(\rm F)}}{T}, \tag{91}$$

where we identified the potential energy variation due to the external conservative force $\Delta E_p^{(\rm F)}$ which completes the total potential energy $\Delta E_p = \Delta E_p^{(\rm rest)} + \Delta E_p^{(\rm F)}$, and:

$$S_\tau^{(\rm F,nc)} \equiv \frac{M}{T}\Big(\frac{g_{12}-g_{21}}{2}\Big)\int_{-\tau/2}^{\tau/2}\delta\mathbf{r}(t)\times\delta\dot{\mathbf{r}}(t)\,dt = \frac{W_\tau^{(\rm nc)}}{T} \tag{92}$$

where we denoted the work of the non-conservative force on the nanowire during time τ by $W_\tau^{(\rm nc)}$. Finally, we obtain remarkable identity:

$$-\Delta E_{\rm kin} - \Delta E_{\rm p} = T S_\tau - W_\tau^{(\rm nc)} \tag{93}$$

The boundary terms $\Delta E_{k,\tau}$ and $\Delta E_{p,\tau}$ will now be neglected. This is justified (according to the argument of [1] and [3]) as they can be expressed as a total time derivative so that the entropy can be redefined without this term, which would obviously modify the value of the entropy at the boundaries without modifying its stationary character. In practice, the statistical average (in the steady state) of the last term $W_\tau^{(\rm nc)}$ will be shown in the next section to be proportional to τ so that the entropy diverges linearly with the duration of the experiment while the boundary terms are bounded in the case of a bounded particle. Therefore:

$$S_\tau \simeq \frac{W_\tau^{(\rm nc)}}{T} \tag{94}$$

Non-conservative force work

The non-conservative forces work $W_\tau^{(\mathrm{nc})}$ given to the nanowire in the interval τ that was identified in the second member of Supplementary Equation 92 is now calculated. First, we will develop the first term of:

$$\int_{-\tau/2}^{\tau/2} \delta\mathbf{r}(t)\times\delta\dot{\mathbf{r}}(t)\,dt=\int_{-\tau/2}^{\tau/2} \delta r_1(t)\delta\dot{r}_2(t)-\delta r_2(t)\delta\dot{r}_1(t)\,dt. \tag{95}$$

into:

$$\int_{-\tau/2}^{\tau/2}\delta r_1(t)\delta\dot{r}_2(t)dt=\int_{-\infty}^{\infty}\frac{d\Omega}{2\pi}\int_{-\infty}^{\infty}\frac{d\Omega'}{2\pi}\,(-i\Omega')\,\delta r_1[\Omega]\delta r_2[\Omega']\times\tau\,\mathrm{sinc}\left(\frac{(\Omega+\Omega')\tau}{2}\right) \tag{96}$$

For Brownian trajectories, we can now realize a statistical average over the steady state denoted by $\langle ...\rangle$ and use the results of the modal development in basis $(1,2)$ which constitutes our model for the whole article: namely, this is equations S17 and S18 applied in the directions of the uncoupled eigenmodes $\beta=0°,90°$, that are now recalled:

Normal mode expansion in the uncoupled eigenmodes basis $(1,2)$

$$\left\{\begin{array}{lcl}\delta r_1[\Omega] &=& \frac{1}{\Xi[\Omega]}\left((\Omega_{2||}^2-\Omega^2-i\Gamma\Omega)\delta F_{\mathrm{th},1}[\Omega]+g_{21}\,\delta F_{\mathrm{th},2}[\Omega]\right)\\ \delta r_2[\Omega] &=& \frac{1}{\Xi[\Omega]}\left(g_{12}\,\delta F_{\mathrm{th},1}[\Omega]+(\Omega_{1||}^2-\Omega^2-i\Gamma\Omega)\delta F_{\mathrm{th},2}[\Omega]\right)\end{array}\right., \tag{97}$$

$$\langle\delta F_{\mathrm{th},i}[\Omega]\delta F_{\mathrm{th},i}[\Omega']\rangle=2\pi\delta(\Omega+\Omega')S_{F_{\mathrm{th},i}}[\Omega]\quad\text{and}\quad\langle\delta F_{\mathrm{th},i}[\Omega]\delta F_{\mathrm{th},j}[\Omega']\rangle=0\quad\text{for}\quad i\neq j.$$

The delta-correlation removes one of the frequency integrals and, after calculation of the term 96 and of its symmetric counterpart in 95, one obtains:

$$\langle W_\tau^{(\mathrm{nc})}\rangle=\tau\times 2k_BT\big(g_{12}-g_{21}\big)^2\int_{-\infty}^{\infty}\frac{d\Omega}{2\pi}\,\frac{\Gamma^2\Omega^2}{\Big((\Omega_-^2-\Omega^2)^2+\Gamma^2\Omega^2\Big)\Big((\Omega_+^2-\Omega^2)^2+\Gamma^2\Omega^2\Big)}. \tag{98}$$

The integrand now clearly displays a product of the axial susceptibilities as defined in Supplementary Equation 62, so that the non-conservative work *rate* can be identified to the first expression given in the main text:

$$\langle\dot{W}^{(\mathrm{nc})}\rangle=2k_BTM^2\,\mathrm{rot}^2(\mathbf{F})\int_{-\infty}^{\infty}\frac{d\Omega}{2\pi}\,(\Gamma\Omega)^2\;|\chi_-^{||}[\Omega]\chi_+^{||}[\Omega]|^2. \tag{99}$$

Finally, according to the definition of the transverse susceptibilities of Supplementary Equation (63), this expression can be simplified into the second expression given in the main text:

$$\langle\dot{W}^{(\mathrm{nc})}\rangle=2k_BT\int_{-\infty}^{\infty}\frac{d\Omega}{2\pi}\,(\Gamma\Omega)^2\times\frac{1}{2}\left(|\chi_-^{\perp}[\Omega]|^2+|\chi_+^{\perp}[\Omega]|^2\right), \tag{100}$$

which was shown in Supplementary Equation 94 to be connected to the constant entropy production rate:

$$\langle\dot{S}\rangle=2k_B\int_{-\infty}^{\infty}\frac{d\Omega}{2\pi}\,(\Gamma\Omega)^2\times\frac{1}{2}\left(|\chi_-^{\perp}[\Omega]|^2+|\chi_+^{\perp}[\Omega]|^2\right). \tag{101}$$

Note that we chose here to artificially symmetrize these expressions of the work and entropy rates using that $\chi_-^{\perp}[\Omega]=\chi_+^{\perp}[\Omega]$.

Conservation of the energy

Calculating in an analogous way the statistical average of the power given by the Langevin force to the nanowire:

$$\begin{aligned}\langle\dot{W}^{(\mathrm{th})}\rangle &\equiv \frac{1}{\tau}\int_{\tau/2}^{\tau/2}\delta\mathbf{F}_{\mathrm{th}}(t)\cdot\delta\dot{\mathbf{r}}(t)\,dt\\ &= 2k_BT\int_{-\infty}^{\infty}\frac{d\Omega}{2\pi}\,(\Gamma\Omega)^2\;\left(|\chi_-^{||}[\Omega]|^2+|\chi_+^{||}[\Omega]|^2\right)\end{aligned} \tag{102}$$

and the statistical average of the work rate algebrically done by the damping force on the nanowire in the interval τ:

$$\begin{aligned}\langle \dot{W}^{(\Gamma)} \rangle &\equiv \frac{1}{\tau}\int_{\tau/2}^{\tau/2} -M\Gamma\delta\dot{\mathbf{r}}(t)\cdot\delta\dot{\mathbf{r}}(t)\,dt \\ &= -2k_BT\int_{-\infty}^{\infty}\frac{d\Omega}{2\pi}\,(\Gamma\Omega)^2\left[\left(|\chi_-^{||}[\Omega]|^2+|\chi_+^{||}[\Omega]|^2\right)+\frac{1}{2}\left(|\chi_-^{\perp}[\Omega]|^2+|\chi_+^{\perp}[\Omega]|^2\right)\right], \end{aligned} \tag{103}$$

one verifies that the power injected by the non-conservative force in the system is fully dissipated along with the (standard) power injected by the Langevin force through the usual dissipation channel in the heat bath. This is a manifestation of the steady-state conjecture:

$$\langle \dot{W}^{(\mathrm{nc})} \rangle + \langle \dot{W}^{(\mathrm{th})} \rangle + \langle \dot{W}^{(\Gamma)} \rangle = 0. \tag{104}$$

Supplementary References

[1] Zamponi, F., Bonetto, F., Cugliandolo, L. F. & Kurchan, J. A fluctuation theorem for non-equilibrium relaxational systems driven by external forces. *J. Stat. Mech.* 09013–09013 (2005).
[2] Kurchan, J. Fluctuation theorem for stochastic dynamics. *J. Phys. A* **31**, 3719–3729 (1998).
[3] Lebowitz, J. L. & Spohn, H. A Gallavotti-Cohen Type Symmetry in the Large Deviation Functional for Stochastic Dynamics. *J. Stat. Phys.* **95**, 333 (1999).
[4] Seifert, U. Entropy production along a stochastic trajectory and an integral fluctuation theorem. *Phys. Rev. Lett.* **95**, 040602 (2005).
[5] Seifert, U. Stochastic thermodynamics, fluctuation theorems and molecular machines. *Rep. Prog. Phys.* **75**, 126001 (2012).
